# Synthesis and crystal structure of 1-{*N*′-[(4-chloro­benzene)­sulfon­yl]carbamimido­yl}-3-phenyl­thio­urea dimethyl sulfoxide monosolvate

**DOI:** 10.1107/S2056989026003245

**Published:** 2026-04-17

**Authors:** Reham A. Mohamed-Ezzat, Galal H. Elgemeie, Peter G. Jones

**Affiliations:** a Chemistry of Natural and Microbial Products Department, Pharmaceutical and Drug Industries Research Institute, National Research Centre, Cairo, Egypt; b Chemistry Department, Faculty of Science, Capital University, Helwan, Egypt; c Institut für Anorganische und Analytische Chemie, Technische Universität Braunschweig, Hagenring 30, D-38106 Braunschweig, Germany; Universität Greifswald, Germany

**Keywords:** crystal structure, guanidine, hydrogen bonds

## Abstract

The title compound, C_14_H_13_ClN_4_O_2_S_2_·C_2_H_6_OS, crystallizes in space group *P*1 with *Z* = 4. Both main (‘parent’) mol­ecules are closely similar except for minor differences in the orientations of the aromatic rings. The bond angles at the nitro­gen atoms of the (S)C—NH—C_guanidine_ moieties are very wide at *ca* 130°. The parent mol­ecules display intra­molecular hydrogen bonds Ph—N—H⋯N—S and C—N(H)—H⋯O=S, and both formula units involve a bifurcated hydrogen bond system from two N—H donors to the oxygen atom of a DMSO mol­ecule; the orientation of the DMSO mol­ecules is however appreciably different. Further classical hydrogen bonds link the residues to form a ribbon parallel to the *b* axis.

## Abstract 2

There are two formula units of the title compound, C_14_H_13_ClN_4_O_2_S_2_·C_2_H_6_OS, in the asymmetric unit. Both main mol­ecules are closely similar except for minor differences in the orientations of the aromatic rings. The bond angles at the nitro­gen atoms of the SC—NH—C_guanidine_ moiety are *ca* 130°. There are several intra- and inter­molecular hydrogen bonds; the latter link the residues to form a ribbon parallel to the *b* axis.

## 1. Chemical context

Sulfonamide and thio­urea are significant pharmacophores for anti­cancer drug development. They have demonstrated potential anti­cancer activity by targeting several cancer-related enzymes and signalling pathways, such as tubulin, cyclin-dependent kinases (CDKs), topoisomerase II, epidermal growth factor (EGFR), BRAF kinase and nucleotide pyrophosphatase/phospho­diesterase (Pingaew *et al.*, 2022 ▸). Sulfonyl thio­urea compounds act as dual inhibitors of type I, II, IX, and XII carbonic anhydrases (Thanh *et al.*, 2025 ▸), and as potential hypoglycaemic agents (Zhang *et al.*, 2009 ▸). The increasing number of established biological effects underlines their potential as a novel generation of therapeutic agents for various diseases.

**Figure 1:**
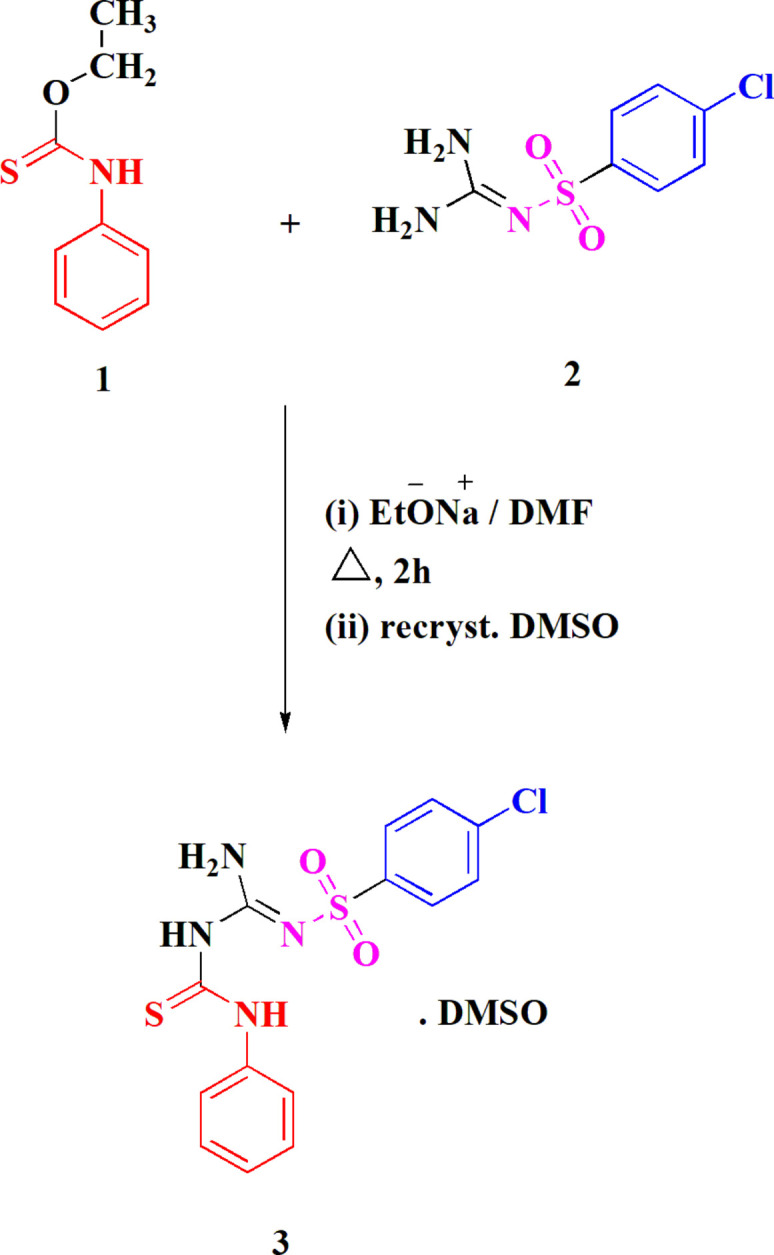
The synthesis of compound **3**.

As a part of our ongoing studies towards developing novel and significant sulfonamide scaffolds (Elgemeie *et al.*, 2015 ▸; Mohamed-Ezzat *et al.*, 2024 ▸, 2025 ▸; Mohamed-Ezzat & Elgemeie, 2024*a* ▸,*b* ▸), we report herein a rational synthetic strategy for the construction of a novel sulfa­thio­urea. The compound combines the pharmacological effects of both thio­urea and sulfonamide moieties, and is therefore likely to exhibit enhanced therapeutic potential, based on previously reported studies on related scaffolds (Pingaew *et al.*, 2022 ▸). We synthesized the sulfa­thio­urea **3**, 1-{*N*′-[(4-chloro­benzene)­sulfon­yl]carbamimido­yl}-3-phenyl­thio­urea dimethyl sulfoxide monosolvate, via the reaction of *o*-ethyl *N*-phenyl­carbamo­thio­ate **1** with *p*-chloro­benzene­sulfonyl­guanidine **2** (Fig. 1 ▸). The ^1^H NMR spectrum of the title compound showed three singlet signals at δ = 8.12, 10.29 and 11.99 ppm, assigned to the NH_2_ and NH protons, in addition to the signals of the aromatic protons. A single-crystal X-ray diffraction analysis of **3**, reported here, was performed to confirm the structure unambiguously; it crystallized from DMSO as the monosolvate **3**·DMSO.
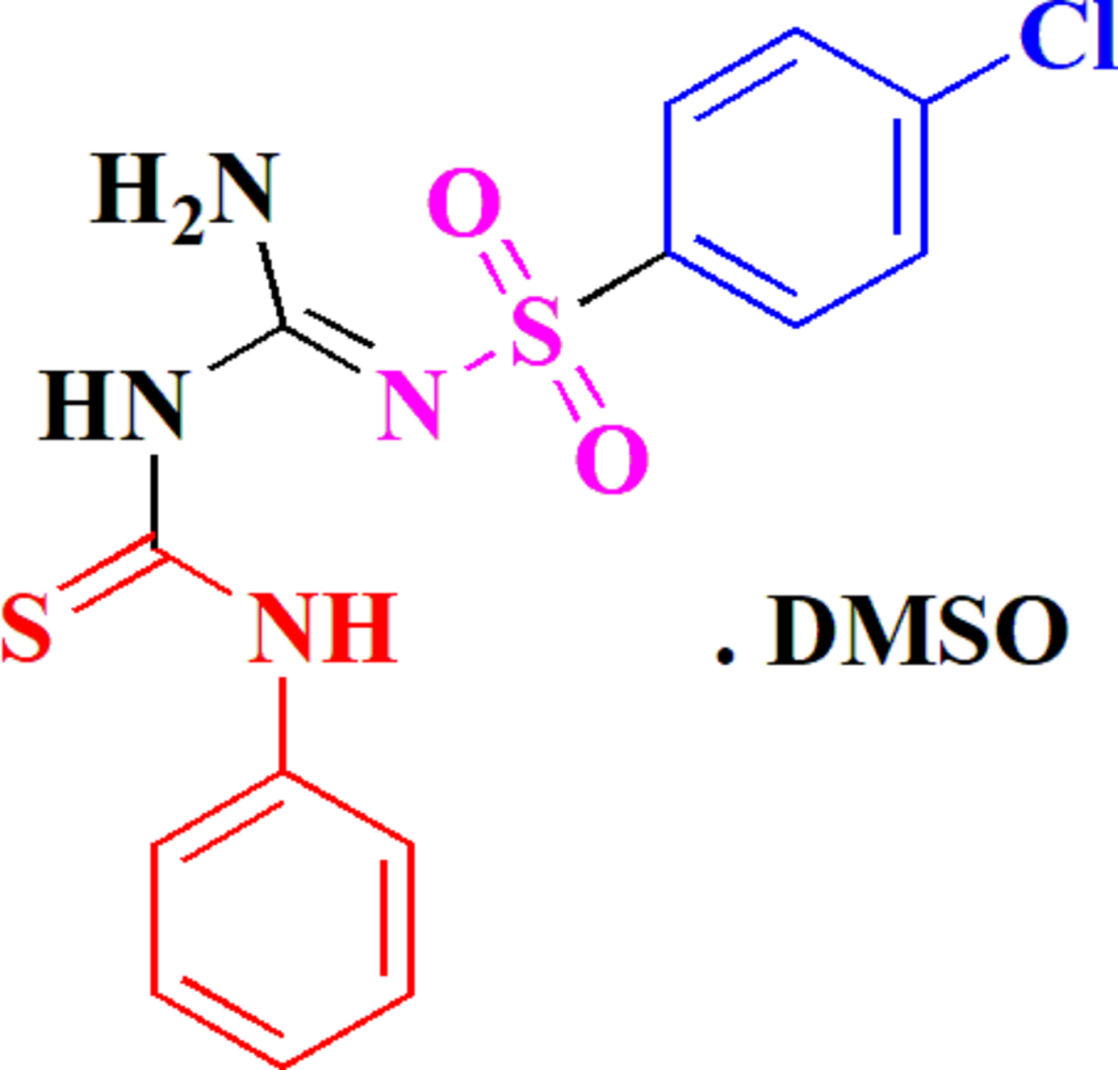


## 2. Structural commentary

**Figure 2:**
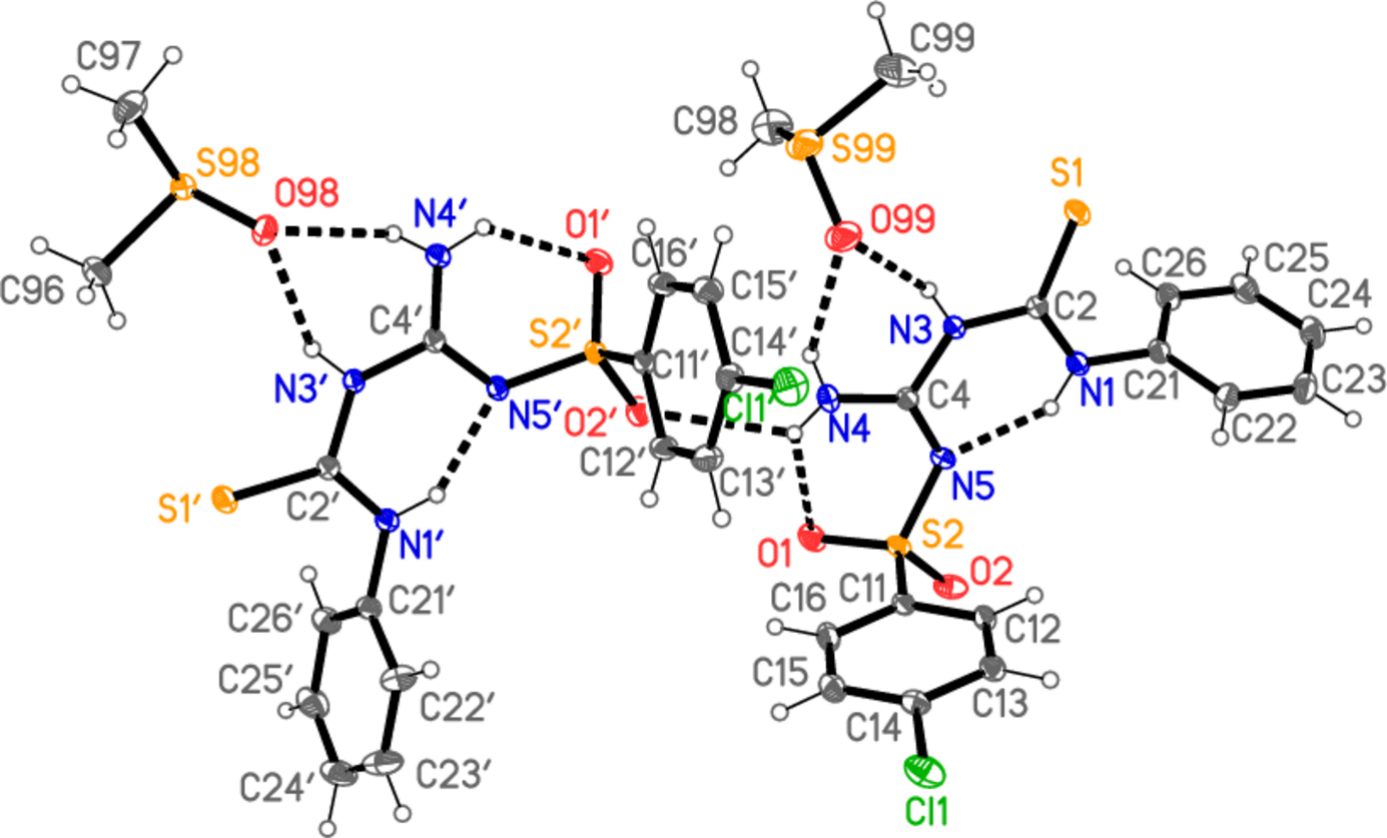
The asymmetric unit of compound **3**. Ellipsoids represent 50% probability levels. Dashed lines indicate hydrogen bonds.

The structure of the title compound is shown in Fig. 2 ▸. Selected geometric parameters are shown in Table 1 ▸. Hydrogen bonds (both intra- and inter­molecular; see also *Supra­molecular features*) are shown in Table 2 ▸. The asymmetric unit contains two formula units; the atom numbering of both main (‘parent’) mol­ecules is the same, with the addition of a prime (′) for the atoms of the second mol­ecule. Both parent mol­ecules display an intra­molecular hydrogen bond from the NH group at N1 to N5; they are linked by the contact N4—H041⋯O2′ (see *Supra­molecular features* for more details of this and of the DMSO inter­actions).

**Figure 3:**
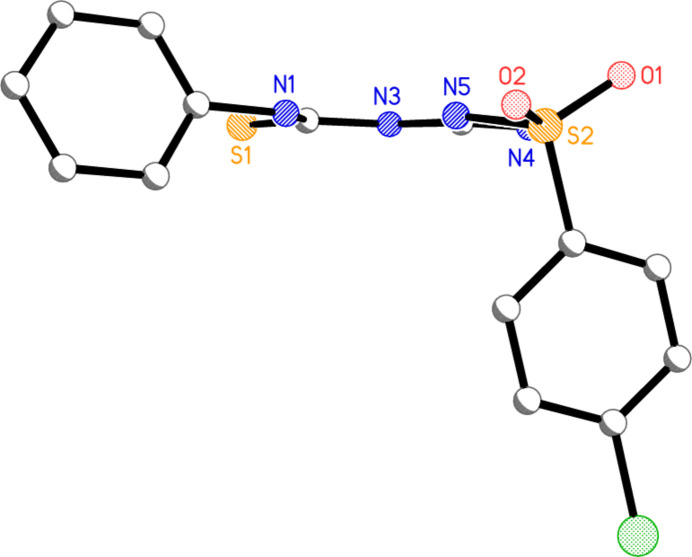
‘Side-on’ view of the first independent mol­ecule of **3** (atom radii are arbitrary; H atoms and solvent are omitted).

Mol­ecular dimensions may be regarded as normal, although the C—N—C angles at N3 and N3′ are, at *ca* 130°, very wide (see also *Database Survey*). Formal bond orders are of limited significance in view of the probably extensive delocalization of multiple bond character. For instance, at the guanidine carbons C4 and C4′, the formal double bonds C4=N5 and C4′=N5′ and the formal single bonds C4—N4 and C4′—N4′ display closely similar lengths. The geometry at all the nitro­gen atoms of the NH and NH_2_ groups is planar, with r.m.s. deviations of 0.004, 0.013, 0.005, 0.004, 0.022 and 0.012 Å at N1, N1′, N3, N3′, N4 and N4′, respectively (calculated for each nitro­gen plus all its three substituents, including the freely refined hydrogen atoms). The central region of both parent mol­ecules is essentially planar, as is shown in the side-view of the first mol­ecule (Fig. 3 ▸; *cf*. torsion angles in Table 1 ▸). The r.m.s. deviation of the nine atoms C2, C4, C21, N1, N3, N4, N5, S1 and O2 is 0.017 and 0.034 Å respectively for the two mol­ecules; S2 and S2′ lie 0.1928 (4) and 0.3024 (4) Å respectively out of the planes thus defined.

**Figure 4:**
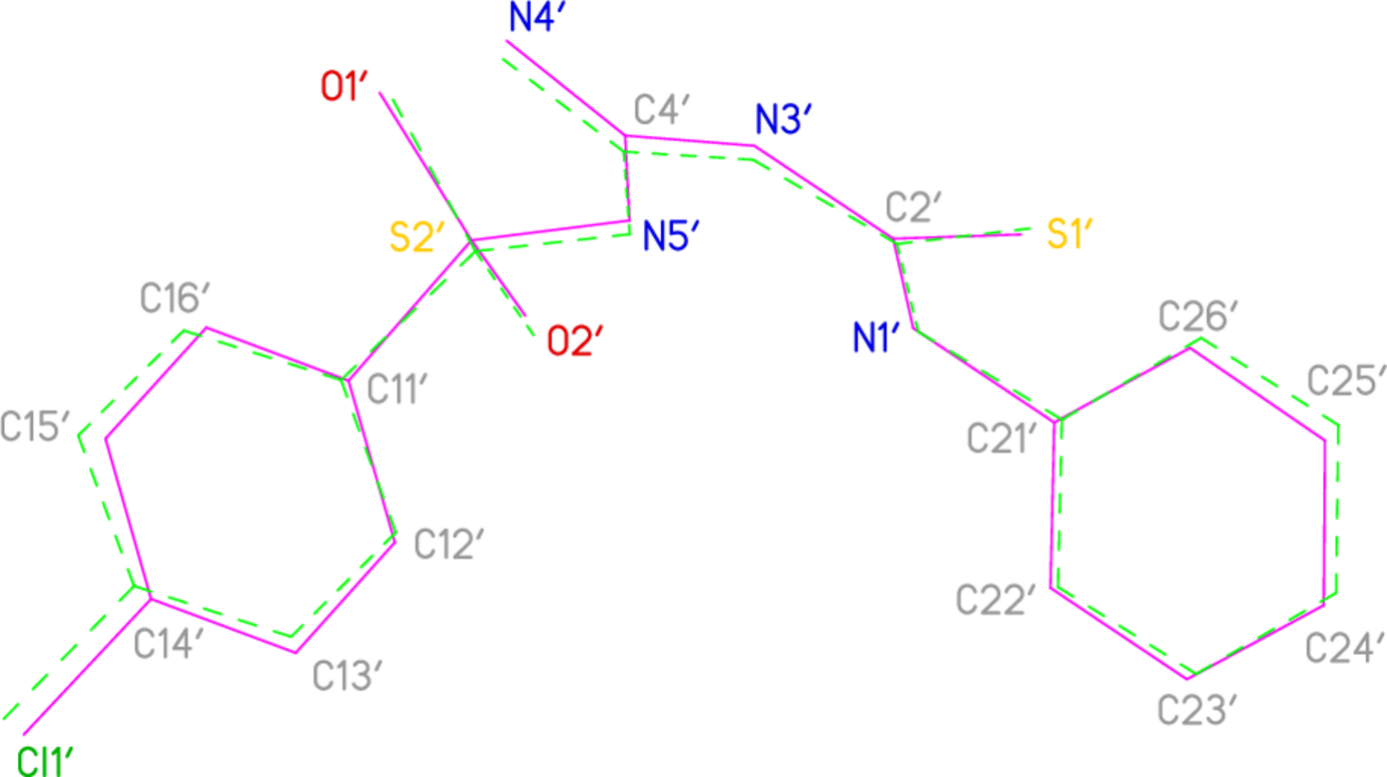
A least-squares fit of the two independent parent mol­ecules of compound **3**. Fitted atoms (of the second parent mol­ecule, dotted bonds) are labelled.

The two parent mol­ecules are closely similar (Table 1 ▸ is arranged to allow ready comparison of both independent mol­ecules). After inversion of one of the mol­ecules, a least-squares fit of all non-hydrogen atoms gave an r.m.s. deviation of 0.21 Å, which decreased to 0.09 Å when the aromatic rings were reduced to just the *ipso* carbon atoms (Fig. 4 ▸).

## 3. Supra­molecular features

**Figure 5:**
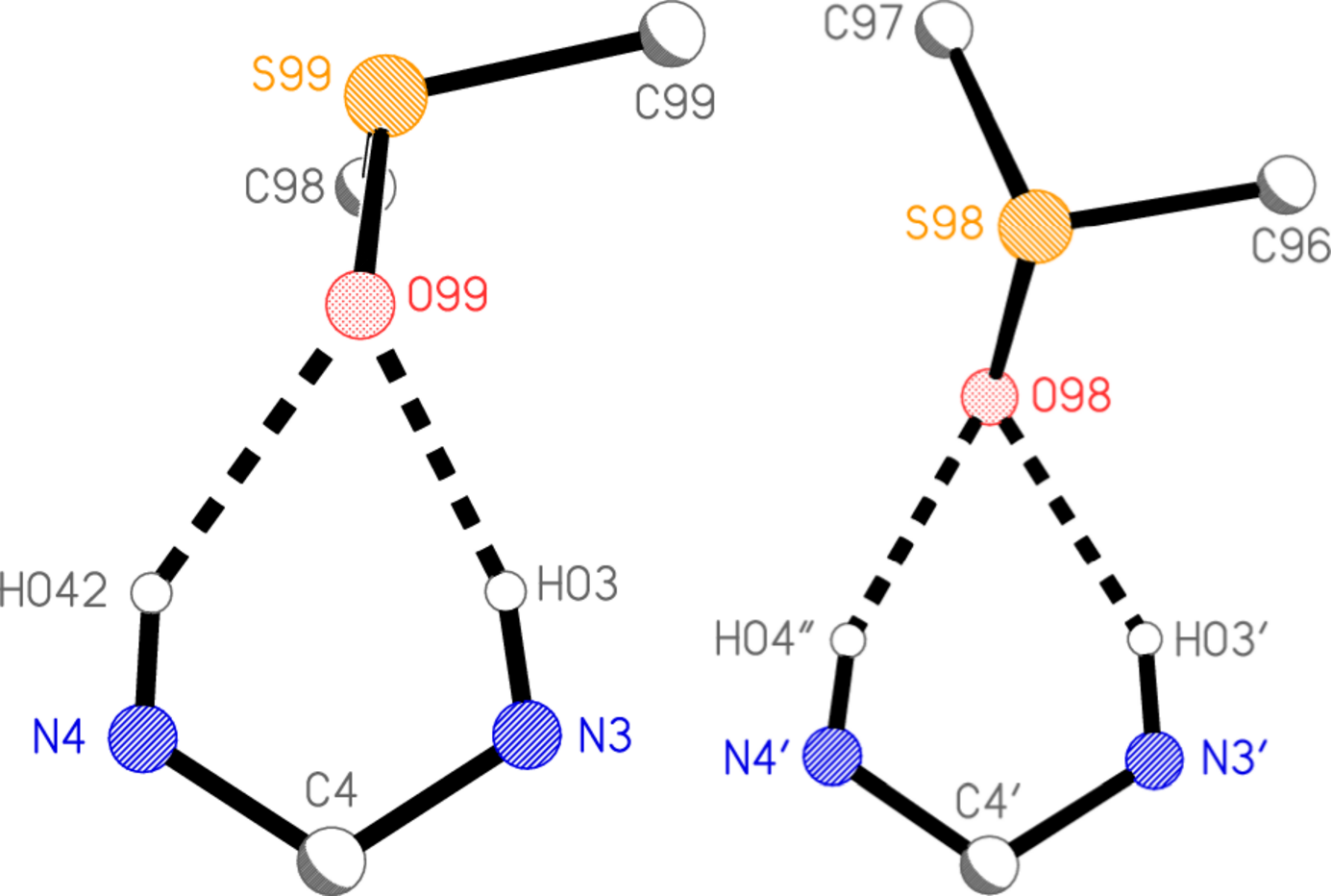
Differing orientations of the DMSO mol­ecules in the two formula units of compound **3** (not to same scale). Radii are arbitrary. Dashed lines indicate hydrogen bonds. The view directions are in both cases perpendicular to the best plane through the selected atoms of the parent mol­ecule.

**Figure 6:**
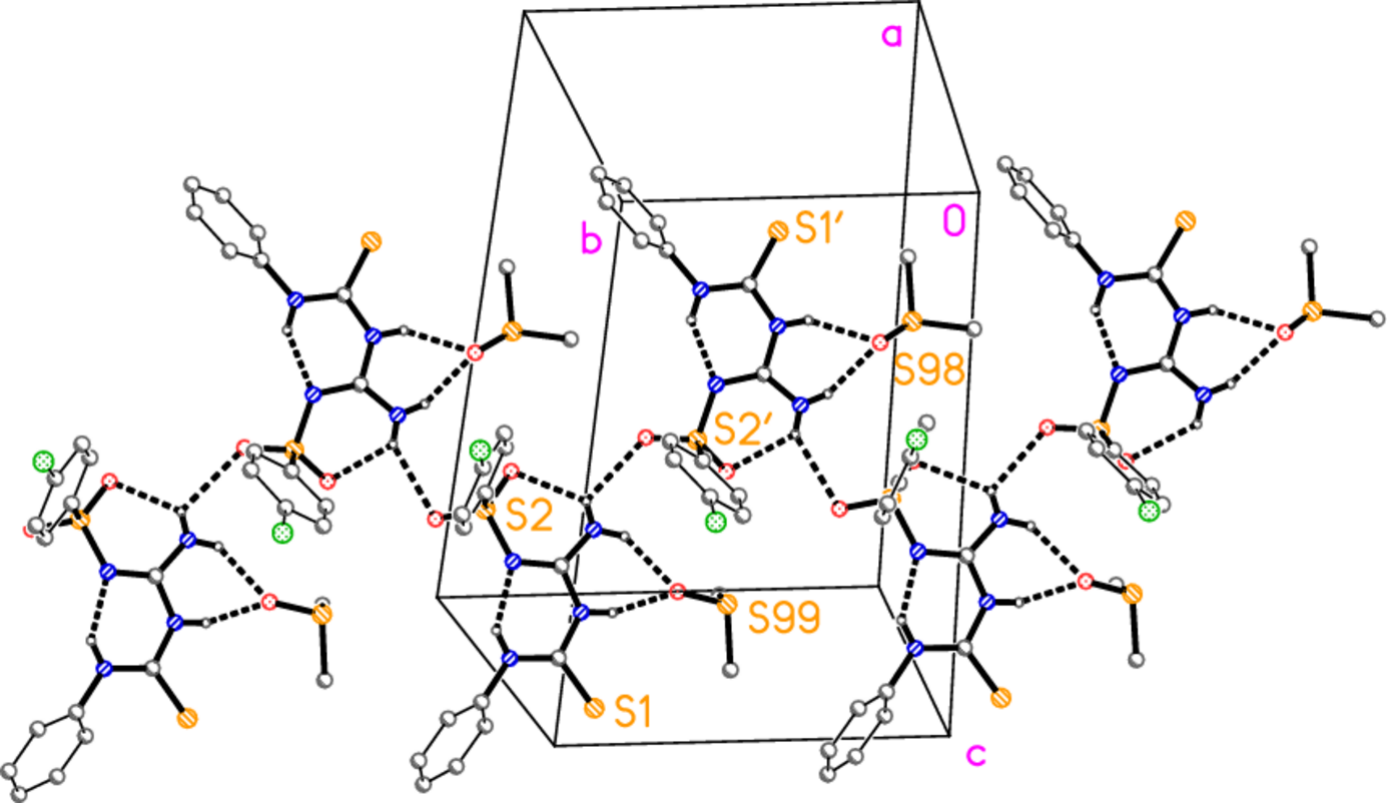
Packing diagram of compound **3** showing the formation of chains of residues parallel to the *b* axis. The view direction is approximately perpendicular to the *bc* plane, but rotated slightly around the horizontal axis for clarity. Dashed lines indicate intra- and inter­molecular hydrogen bonds. The labelled sulfur atoms correspond to the asymmetric unit.

Hydrogen bonds are listed in Table 2 ▸, which for completeness includes some probable ‘weak’ hydrogen bonds of the form C—H⋯O and C—H⋯S. These are, however, not discussed here, because we regard the classical hydrogen bonds as more relevant. In each formula unit, the DMSO oxygen atom accepts hydrogen bonds from the NH group at N3 and one hydrogen atom of the NH_2_ group at N4, thus forming a bifurcated hydrogen bond system. The DMSO mol­ecules are however differently oriented with respect to their parent mol­ecules (Fig. 5 ▸), as seen by torsion angle pairs such as H03⋯O99—S99—C98 52.08 (6) *vs* H03′⋯O98—S98—C97 160.3 (4)°. Also within the asymmetric unit, the two parent mol­ecules are connected by the contact N4—H041⋯O2′ (Fig. 2 ▸), which forms one branch of a three-centre system. The analogous contact N4′⋯H04′⋯O2(*x*, −1 + *y*, *z*) links the parent mol­ecules further, resulting in chains parallel to the *b* axis (Fig. 6 ▸).

## 4. Database survey

Searches were conducted using CSD Version 6.00 (update August 2025; Groom *et al.*, 2016 ▸) and the ConQuest routine (Bruno *et al.*, 2002 ▸), Version 2025.2.0.

The central feature of compound **3** is the acyclic sequence of atoms N–C–N–C–N–S, which connects the aromatic rings. A search for the fragment Ar–N^3^–C^3^–N^3^–C^3^–N^any^, where the superscripts indicate coordination number, all bonds are acyclic and Ar is a phenyl group with any substituents, and with no restriction on bond orders, gave 64 hits. Extending the search fragment to Ar–N^3^–C^3^(–S^1^)–N^3^–C^3^–N^any^, as in **3**, restricted the number of hits to six: 1-(2-chloro­phen­yl)-3-[(phen­yl)(4-tolyl­imino)­meth­yl]thio­urea (refcode ADAVOR; Xing & Zhao, 2006*a* ▸), 5-benzoyl-1-phenyl-thio­biuret (BAQJUY; Reinke *et al.*, 1999 ▸), 1-[(phen­yl)(*p*-tolyl­imino)­meth­yl]-3-(*p*-tol­yl)thio­urea [IDOFUD; Xing & Zhao, 2006*b* ▸), 1-[(phen­yl)(*p*-tolyl­imino)­meth­yl]-3-(*o*-tol­yl)thio­urea [MET­TIP; Song *et al.*, 2007 ▸), *N*′,2-di­benzyl­idene-*N*-(phenyl­carbamo­thio­yl)hydrazine-1-carbohydrazonamide (PEQGEB; Tapera *et al.*, 2022 ▸) and 3-{[(di­ethyl­carbamo­yl)carbamo­thio­yl]amino}­benzoic acid monohydrate [TAVCOM; Khan *et al.*, 2022 ▸). Similarly to **3**, all of these structures have a wide C—N—C angle at the atom corresponding to N3 in **3** (see above); the values range from 127.9 to 130.6°, average 129.6°. Alternatively, extending the first fragment to Ar–N^3^–C^3^–N^3^–C^3^–N^any^–S^any^, as in **3**, gave just one hit, namely *N*-{[(4-meth­oxy­phen­yl)carbamo­yl]carbamo­yl}-4-methyl­benzene-1-sulfonamide (ANILOB; Mahapatra *et al.*, 2021 ▸).

## 5. Synthesis and crystallization

A mixture of *o*-ethyl *N*-phenyl­carbamo­thio­ate and *p*-chloro­benzene­sulfonyl­guanidine (0.01 mol) **1** was refluxed in dry di­methyl­formamide (10 ml) containing sodium ethoxide (0.01 mol) for 2 h. After completion of the reaction, the mixture was poured into ice–water and neutralized with hydro­chloric acid. The precipitate thus formed was collected by filtration, washed with water, dried, and then recrystallized from dimethyl sulfoxide, affording compound **3** as pale-yellow crystals (individual crystals viewed under the microscope were effectively colourless) in 65% yield, m.p. 464–465 K; ^1^H NMR (500 MHz, DMSO-*d_6_*): δ (ppm) 7.21–7.22 (*d*, 1H, Ar-H), 7.36 (*m*, 2H, Ar-H), 7.46–7.47 (*d*, 2H, Hz, Ar-H), 7.63 (*d*, 2H, *J* = 10 Hz, Ar-H), 7.91 (*d*, 2H, *J* = 10 Hz, Ar-H), 8.12 (*s*, 1H, NH), 10.29 (*s*, 2H, NH_2_), 11.99 (*s*, 1H, NH); ^13^C NMR (500 MHz, DMSO-*d*_6_): δ (ppm) 124.22, 126.88, 128.05, 128.63, 129.10, 129.35, 129.75, 129.87, 130.41, 138.02, 153.18, 177.98. Analysis: calculated for C_14_H_13_ClN_4_O_2_S_2_ (368.86): C 45.59, H 3.55, Cl 9.61, N 15.19, S 17.39. Found: C 45.51, H 3.48, Cl 9.58, N 15.13, S 17.37%.

## 6. Refinement

Details of data collection and structure refinement are summarized in Table 3 ▸. The numbering N1–C2–N3–C4–N5 was chosen to accentuate the importance of this acyclic atom sequence in the centre of the parent molecule(s). It should be noted, however, that this numbering is not consistent with the IUPAC name, which has a sulfonyl-carbamimidoyl group at N1 and a phenyl group at N3 (these nitrogen atoms are N3 and N1 respectively in our numbering). The hydrogen atoms of the NH and NH_2_ groups were refined freely. The methyl groups were refined as idealized rigid groups with C—H = 0.98 Å, H—C—H = 109.5°, allowed to rotate but not tip (AFIX 137). Other hydrogen atoms were included using a riding model starting from calculated positions with C*sp*^2^—H = 0.95 Å. The *U*_iso_(H) values were fixed at 1.5 × *U*_eq_ of the parent carbon atoms for the methyl groups and 1.2 × *U*_eq_ for the other hydrogens. Seven badly-fitting reflections with Δ/σ > 9 were omitted from the refinement.

## Supplementary Material

Crystal structure: contains datablock(s) I, global. DOI: 10.1107/S2056989026003245/yz2076sup1.cif

Structure factors: contains datablock(s) I. DOI: 10.1107/S2056989026003245/yz2076Isup2.hkl

Supporting information file. DOI: 10.1107/S2056989026003245/yz2076Isup3.cml

CCDC reference: 2541315

Additional supporting information:  crystallographic information; 3D view; checkCIF report

## Figures and Tables

**Table 1:** Selected geometric parameters (Å, °)

N1—C2	1.3338 (9)	N1′—C2′	1.3315 (9)
N1—C21	1.4293 (9)	N1′—C21′	1.4305 (9)
C2—N3	1.3881 (9)	C2′—N3′	1.3871 (9)
C2—S1	1.6746 (7)	C2′—S1′	1.6743 (7)
N3—C4	1.3770 (9)	N3′—C4′	1.3780 (9)
C4—N5	1.3235 (9)	C4′—N5′	1.3319 (8)
C4—N4	1.3333 (9)	C4′—N4′	1.3293 (9)
N5—S2	1.6136 (6)	N5′—S2′	1.6100 (6)
S2—O2	1.4400 (6)	S2′—O2′	1.4425 (5)
S2—O1	1.4463 (6)	S2′—O1′	1.4459 (6)
S2—C11	1.7660 (7)	S2′—C11′	1.7696 (7)
			
C2—N1—C21	124.14 (6)	C2′—N1′—C21′	123.45 (6)
N1—C2—N3	117.33 (6)	N1′—C2′—N3′	117.29 (6)
N1—C2—S1	125.26 (5)	N1′—C2′—S1′	124.72 (5)
N3—C2—S1	117.41 (5)	N3′—C2′—S1′	117.97 (5)
C4—N3—C2	129.78 (6)	C4′—N3′—C2′	130.12 (6)
N4—C4—N5	127.20 (6)	N4′—C4′—N5′	127.34 (6)
N5—C4—N3	118.66 (6)	N5′—C4′—N3′	118.47 (6)
N4—C4—N3	114.13 (6)	N4′—C4′—N3′	114.18 (6)
C4—N5—S2	122.03 (5)	C4′—N5′—S2′	121.16 (5)
			
C21—N1—C2—N3	177.44 (6)	C21′—N1′—C2′—N3′	−176.36 (6)
N1—C2—N3—C4	0.07 (11)	N1′—C2′—N3′—C4′	−3.68 (11)
C2—N3—C4—N5	0.21 (11)	C2′—N3′—C4′—N5′	2.99 (11)
N3—C4—N5—S2	171.61 (5)	N3′—C4′—N5′—S2′	−168.80 (5)
C4—N5—S2—C11	−71.14 (6)	C4′—N5′—S2′—C11′	75.15 (6)
N5—S2—C11—C16	125.11 (6)	N5′—S2′—C11′—C16′	−110.01 (6)
N5—S2—C11—C12	−56.63 (6)	N5′—S2′—C11′—C12′	70.45 (6)
C2—N1—C21—C22	110.41 (8)	C2′—N1′—C21′—C22′	−101.83 (9)
C2—N1—C21—C26	−72.77 (9)	C2′—N1′—C21′—C26′	81.93 (9)

**Table 2:** Hydrogen-bond geometry (Å, °)

*D*—H⋯*A*	*D*—H	H⋯*A*	*D*⋯*A*	*D*—H⋯*A*
N1—H01⋯N5	0.848 (15)	1.929 (15)	2.6375 (8)	140.3 (13)
N3—H03⋯O99	0.860 (15)	1.902 (15)	2.7300 (9)	161.2 (15)
N4—H041⋯O1	0.809 (16)	2.341 (16)	2.9130 (10)	128.4 (13)
N4—H041⋯O2′	0.809 (16)	2.478 (15)	2.9982 (8)	123.2 (13)
N4—H042⋯O99	0.861 (15)	2.112 (15)	2.8713 (10)	146.7 (13)
N1′—H01′⋯N5′	0.879 (15)	1.902 (15)	2.6458 (8)	141.3 (14)
N3′—H03′⋯O98	0.850 (13)	2.060 (13)	2.8379 (8)	151.8 (12)
N4′—H04′⋯O2^i^	0.829 (15)	2.402 (15)	3.0350 (9)	133.8 (13)
N4′—H04′⋯O1′	0.829 (15)	2.220 (15)	2.8324 (9)	130.9 (13)
N4′—H04"⋯O98	0.803 (14)	2.035 (14)	2.7903 (9)	156.7 (14)
C15—H15⋯S1′^ii^	0.95	2.80	3.6671 (8)	153
C98—H98*C*⋯O1′	0.98	2.66	3.4937 (15)	144
C99—H99*C*⋯S1	0.98	3.01	3.8460 (13)	144
C16′—H16′⋯O2^i^	0.95	2.45	3.3273 (9)	153
C26′—H26′⋯O2^iii^	0.95	2.55	3.2038 (9)	127
C96—H96*B*⋯O1^iv^	0.98	2.60	3.2186 (11)	121
C97—H97*C*⋯O2′^i^	0.98	2.56	3.4130 (11)	146

Symmetry codes: (i) 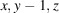; (ii) 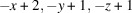; (iii) 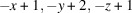; (iv) 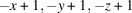.

**Table 3:** Experimental details

Crystal data	
Chemical formula	C_14_H_13_ClN_4_O_2_S_2_·C_2_H_6_OS
*M*_r_	446.98
Crystal system, space group	Triclinic, *P* 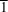
Temperature (K)	100
*a*, *b*, *c* (Å)	10.5374 (2), 11.04935 (16), 18.2768 (4)
α, β, γ (°)	83.5414 (14), 86.6369 (18), 76.8708 (16)
*V* (Å^3^)	2058.02 (7)
*Z*	4
Radiation type	Mo *K*α
μ (mm^−1^)	0.51
Crystal size (mm)	0.15 × 0.15 × 0.10

Data collection	
Diffractometer	XtaLAB Synergy
Absorption correction	Multi-scan (*CrysAlis PRO*; Rigaku OD, 2025 ▸)
*T*_min_, *T*_max_	0.881, 1.000
No. of measured, independent and observed [*I* > 2σ(*I*)] reflections	260714, 26901, 21151
*R*_int_	0.038
θ values (°)	θ_max_ = 41.3, θ_min_ = 2.1
(sin θ/λ)_max_ (Å^−1^)	0.928

Refinement	
*R*[*F*^2^ > 2σ(*F*^2^)], *wR*(*F*^2^), *S*	0.032, 0.094, 1.05
No. of reflections	26901
No. of parameters	523
H-atom treatment	H atoms treated by a mixture of independent and constrained refinement
Δρ_max_, Δρ_min_ (e Å^−3^)	1.02, −0.74

Computer programs: *CrysAlis PRO* (Rigaku OD, 2025 ▸), *SHELXT* (Sheldrick, 2015*a* ▸), *SHELXL2019/3* (Sheldrick, 2015*b* ▸), *XP* (Bruker, 1998 ▸) and *publCIF* (Westrip, 2010 ▸).

## supplementary crystallographic information

### 1-{N'-[(4-Chlorobenzene)sulfonyl]carbamimidoyl}-3-phenylthiourea dimethyl sulfoxide monosolvate Crystal data

**Table:**

C_14_H_13_ClN_4_O_2_S_2_·C_2_H_6_OS	*Z* = 4
*M**_r_* = 446.98	*F*(000) = 928
Triclinic, *P*1	*D*_x_ = 1.443 Mg m^−^^3^
*a* = 10.5374 (2) Å	Mo *K*α radiation, λ = 0.71073 Å
*b* = 11.04935 (16) Å	Cell parameters from 117740 reflections
*c* = 18.2768 (4) Å	θ = 2.1–41.4°
α = 83.5414 (14)°	µ = 0.51 mm^−^^1^
β = 86.6369 (18)°	*T* = 100 K
γ = 76.8708 (16)°	Tablet, colourless
*V* = 2058.02 (7) Å^3^	0.15 × 0.15 × 0.10 mm

### 1-{N'-[(4-Chlorobenzene)sulfonyl]carbamimidoyl}-3-phenylthiourea dimethyl sulfoxide monosolvate Data collection

**Table:**

XtaLAB Synergy diffractometer	26901 independent reflections
Radiation source: micro-focus sealed X-ray tube, PhotonJet (Mo) X-ray Source	21151 reflections with *I* > 2σ(*I*)
Mirror monochromator	*R*_int_ = 0.038
Detector resolution: 10.0000 pixels mm^-1^	θ_max_ = 41.3°, θ_min_ = 2.1°
ω scans	*h* = −19→19
Absorption correction: multi-scan (CrysAlisPro; Rigaku OD, 2025)	*k* = −20→20
*T*_min_ = 0.881, *T*_max_ = 1.000	*l* = −33→33
260714 measured reflections	

### 1-{N'-[(4-Chlorobenzene)sulfonyl]carbamimidoyl}-3-phenylthiourea dimethyl sulfoxide monosolvate Refinement

**Table:**

Refinement on *F*^2^	Primary atom site location: dual
Least-squares matrix: full	Hydrogen site location: mixed
*R*[*F*^2^ > 2σ(*F*^2^)] = 0.032	H atoms treated by a mixture of independent and constrained refinement
*wR*(*F*^2^) = 0.094	*w* = 1/[σ^2^(*F*_o_^2^) + (0.049*P*)^2^ + 0.3254*P*] where *P* = (*F*_o_^2^ + 2*F*_c_^2^)/3
*S* = 1.05	(Δ/σ)_max_ = 0.006
26901 reflections	Δρ_max_ = 1.02 e Å^−^^3^
523 parameters	Δρ_min_ = −0.74 e Å^−^^3^
0 restraints	

### 1-{N'-[(4-Chlorobenzene)sulfonyl]carbamimidoyl}-3-phenylthiourea dimethyl sulfoxide monosolvate Special details

**Table:**

Refinement. Hydrogen atoms bonded to nitrogen were refined freely.Seven reflections with delta/sigma > 9 were omitted from the refinement.

### 1-{N'-[(4-Chlorobenzene)sulfonyl]carbamimidoyl}-3-phenylthiourea dimethyl sulfoxide monosolvate Fractional atomic coordinates and isotropic or equivalent isotropic displacement parameters (Å^2^)

**Table:**

	*x*	*y*	*z*	*U*_iso_*/*U*_eq_	
N1	0.31930 (6)	1.03887 (6)	0.92813 (3)	0.01559 (9)	
H01	0.3525 (14)	1.0708 (13)	0.8894 (8)	0.031 (3)*	
C2	0.32859 (6)	0.91596 (6)	0.93160 (4)	0.01332 (9)	
N3	0.38850 (6)	0.85538 (5)	0.87194 (3)	0.01388 (9)	
H03	0.3959 (14)	0.7758 (14)	0.8759 (8)	0.034 (4)*	
C4	0.44195 (6)	0.90281 (6)	0.80751 (4)	0.01371 (9)	
N4	0.49276 (7)	0.81749 (7)	0.76154 (4)	0.02005 (11)	
H041	0.5154 (15)	0.8379 (14)	0.7198 (9)	0.035 (4)*	
H042	0.4895 (14)	0.7406 (14)	0.7735 (8)	0.031 (3)*	
N5	0.43927 (6)	1.02387 (6)	0.79761 (3)	0.01477 (9)	
S1	0.27497 (2)	0.82873 (2)	1.00241 (2)	0.01937 (3)	
S2	0.51613 (2)	1.08448 (2)	0.72928 (2)	0.01470 (3)	
O1	0.49803 (6)	1.03957 (7)	0.66014 (3)	0.02306 (11)	
O2	0.47973 (6)	1.21725 (5)	0.73371 (3)	0.02007 (10)	
C11	0.68286 (6)	1.03276 (6)	0.74925 (4)	0.01364 (9)	
C12	0.72434 (6)	1.05898 (6)	0.81573 (4)	0.01395 (9)	
H12	0.663416	1.101960	0.849629	0.017*	
C13	0.85527 (7)	1.02169 (7)	0.83178 (4)	0.01541 (10)	
H13	0.885400	1.039030	0.876615	0.018*	
C14	0.94186 (7)	0.95837 (6)	0.78103 (4)	0.01593 (10)	
C15	0.90193 (7)	0.93258 (8)	0.71470 (4)	0.01975 (12)	
H15	0.963154	0.889817	0.680859	0.024*	
C16	0.76992 (7)	0.97061 (8)	0.69848 (4)	0.01884 (12)	
H16	0.740107	0.954184	0.653335	0.023*	
Cl1	1.10606 (2)	0.91336 (2)	0.80150 (2)	0.02448 (4)	
C21	0.25599 (7)	1.11537 (6)	0.98356 (4)	0.01457 (10)	
C22	0.33069 (8)	1.16617 (7)	1.02675 (4)	0.01902 (12)	
H22	0.423002	1.147621	1.020637	0.023*	
C23	0.26922 (9)	1.24457 (8)	1.07913 (5)	0.02266 (14)	
H23	0.319666	1.279084	1.109153	0.027*	
C24	0.13391 (9)	1.27216 (7)	1.08735 (4)	0.02216 (13)	
H24	0.092018	1.325405	1.123099	0.027*	
C25	0.05982 (8)	1.22191 (7)	1.04331 (4)	0.02075 (13)	
H25	−0.032577	1.241464	1.048808	0.025*	
C26	0.12064 (7)	1.14317 (7)	0.99127 (4)	0.01753 (11)	
H26	0.070198	1.108660	0.961242	0.021*	
S99	0.37883 (3)	0.49924 (2)	0.86206 (2)	0.03091 (5)	
C98	0.23684 (13)	0.55769 (13)	0.80985 (8)	0.0418 (3)	
H98A	0.185638	0.633627	0.829291	0.063*	
H98B	0.184223	0.494517	0.813251	0.063*	
H98C	0.262327	0.577151	0.758195	0.063*	
C99	0.30180 (16)	0.49498 (12)	0.95193 (7)	0.0461 (3)	
H99A	0.368384	0.466001	0.989070	0.069*	
H99B	0.242566	0.437670	0.955436	0.069*	
H99C	0.252277	0.578919	0.960504	0.069*	
O99	0.44903 (7)	0.60539 (6)	0.85736 (5)	0.03027 (14)	
N1'	0.79393 (6)	0.53267 (5)	0.44404 (3)	0.01463 (9)	
H01'	0.7374 (15)	0.5640 (14)	0.4781 (8)	0.034 (4)*	
C2'	0.80527 (6)	0.41096 (6)	0.44047 (4)	0.01364 (9)	
N3'	0.73079 (6)	0.35016 (5)	0.49062 (3)	0.01471 (9)	
H03'	0.7405 (13)	0.2734 (13)	0.4846 (7)	0.023 (3)*	
C4'	0.64878 (6)	0.39433 (6)	0.54792 (3)	0.01291 (9)	
N4'	0.59848 (7)	0.30770 (6)	0.58827 (4)	0.01752 (10)	
H04'	0.5455 (14)	0.3280 (14)	0.6225 (8)	0.031 (3)*	
H04"	0.6163 (13)	0.2386 (13)	0.5753 (8)	0.026 (3)*	
N5'	0.62989 (6)	0.51476 (5)	0.55793 (3)	0.01396 (9)	
S1'	0.90544 (2)	0.32600 (2)	0.38144 (2)	0.02132 (4)	
S2'	0.55719 (2)	0.56779 (2)	0.63152 (2)	0.01242 (3)	
O1'	0.44615 (5)	0.51479 (5)	0.65487 (3)	0.01729 (9)	
O2'	0.53538 (6)	0.70203 (5)	0.61877 (3)	0.01771 (9)	
C11'	0.67353 (6)	0.51718 (6)	0.70083 (3)	0.01282 (9)	
C12'	0.78215 (7)	0.57014 (7)	0.69930 (4)	0.01622 (10)	
H12'	0.794950	0.631466	0.660493	0.019*	
C13'	0.87139 (7)	0.53248 (7)	0.75497 (4)	0.01819 (11)	
H13'	0.946353	0.566795	0.754507	0.022*	
C14'	0.84871 (7)	0.44336 (7)	0.81147 (4)	0.01665 (11)	
Cl1'	0.95810 (2)	0.39497 (2)	0.88224 (2)	0.02390 (4)	
C15'	0.74242 (8)	0.38906 (7)	0.81280 (4)	0.01825 (11)	
H15'	0.729769	0.327788	0.851665	0.022*	
C16'	0.65419 (7)	0.42553 (6)	0.75632 (4)	0.01617 (10)	
H16'	0.581529	0.388154	0.755719	0.019*	
C21'	0.85893 (6)	0.60876 (6)	0.39335 (4)	0.01387 (9)	
C22'	0.97036 (8)	0.64143 (7)	0.41413 (5)	0.02151 (13)	
H22'	1.005542	0.611002	0.461077	0.026*	
C23'	1.02987 (9)	0.71940 (8)	0.36523 (6)	0.02740 (17)	
H23'	1.106469	0.741996	0.378643	0.033*	
C24'	0.97743 (9)	0.76406 (7)	0.29699 (6)	0.02667 (17)	
H24'	1.019186	0.815964	0.263436	0.032*	
C25'	0.86428 (9)	0.73332 (8)	0.27748 (5)	0.02443 (15)	
H25'	0.827524	0.765998	0.231186	0.029*	
C26'	0.80460 (7)	0.65473 (7)	0.32561 (4)	0.01802 (11)	
H26'	0.727596	0.632756	0.312296	0.022*	
S98	0.65810 (2)	0.03146 (2)	0.46454 (2)	0.01620 (3)	
C96	0.76467 (10)	0.03143 (10)	0.38501 (5)	0.02742 (17)	
H96A	0.756387	0.117163	0.361872	0.041*	
H96B	0.741311	−0.020092	0.349917	0.041*	
H96C	0.854850	−0.002787	0.399549	0.041*	
C97	0.70167 (9)	−0.13112 (7)	0.49503 (6)	0.02628 (16)	
H97A	0.795759	−0.155979	0.502434	0.039*	
H97B	0.678605	−0.179025	0.457828	0.039*	
H97C	0.654597	−0.147714	0.541586	0.039*	
O98	0.71669 (6)	0.09566 (5)	0.51927 (3)	0.02093 (10)	

### 1-{N'-[(4-Chlorobenzene)sulfonyl]carbamimidoyl}-3-phenylthiourea dimethyl sulfoxide monosolvate Atomic displacement parameters (Å^2^)

**Table:**

	*U*^11^	*U*^22^	*U*^33^	*U*^12^	*U*^13^	*U*^23^
N1	0.0194 (2)	0.0140 (2)	0.0135 (2)	−0.00474 (18)	0.00416 (18)	−0.00262 (16)
C2	0.0145 (2)	0.0148 (2)	0.0113 (2)	−0.00447 (18)	0.00075 (18)	−0.00195 (17)
N3	0.0161 (2)	0.0140 (2)	0.0122 (2)	−0.00494 (17)	0.00301 (17)	−0.00258 (16)
C4	0.0133 (2)	0.0172 (2)	0.0116 (2)	−0.00525 (19)	0.00109 (18)	−0.00268 (18)
N4	0.0263 (3)	0.0208 (3)	0.0147 (2)	−0.0080 (2)	0.0066 (2)	−0.0068 (2)
N5	0.0154 (2)	0.0164 (2)	0.0129 (2)	−0.00569 (17)	0.00253 (17)	−0.00047 (17)
S1	0.02798 (9)	0.01764 (7)	0.01293 (7)	−0.00793 (6)	0.00558 (6)	−0.00081 (5)
S2	0.01345 (6)	0.02001 (7)	0.01104 (6)	−0.00626 (5)	−0.00035 (5)	0.00184 (5)
O1	0.0217 (2)	0.0395 (3)	0.0113 (2)	−0.0142 (2)	−0.00139 (18)	−0.0012 (2)
O2	0.0170 (2)	0.0183 (2)	0.0233 (3)	−0.00439 (17)	−0.00108 (18)	0.00631 (18)
C11	0.0135 (2)	0.0168 (2)	0.0111 (2)	−0.00457 (18)	0.00112 (18)	−0.00161 (18)
C12	0.0145 (2)	0.0161 (2)	0.0110 (2)	−0.00310 (19)	0.00065 (18)	−0.00192 (18)
C13	0.0151 (2)	0.0181 (3)	0.0128 (2)	−0.0035 (2)	−0.00048 (19)	−0.00043 (19)
C14	0.0130 (2)	0.0161 (2)	0.0175 (3)	−0.00254 (19)	0.0009 (2)	0.0012 (2)
C15	0.0170 (3)	0.0230 (3)	0.0190 (3)	−0.0032 (2)	0.0044 (2)	−0.0066 (2)
C16	0.0178 (3)	0.0254 (3)	0.0145 (3)	−0.0054 (2)	0.0024 (2)	−0.0073 (2)
Cl1	0.01335 (7)	0.03095 (9)	0.02539 (9)	−0.00067 (6)	−0.00021 (6)	0.00411 (7)
C21	0.0169 (3)	0.0139 (2)	0.0123 (2)	−0.00237 (19)	0.00094 (19)	−0.00211 (18)
C22	0.0187 (3)	0.0195 (3)	0.0196 (3)	−0.0040 (2)	−0.0015 (2)	−0.0049 (2)
C23	0.0304 (4)	0.0199 (3)	0.0187 (3)	−0.0053 (3)	−0.0023 (3)	−0.0066 (2)
C24	0.0308 (4)	0.0164 (3)	0.0166 (3)	0.0001 (2)	0.0032 (3)	−0.0032 (2)
C25	0.0196 (3)	0.0202 (3)	0.0189 (3)	0.0017 (2)	0.0032 (2)	−0.0013 (2)
C26	0.0160 (3)	0.0198 (3)	0.0158 (3)	−0.0021 (2)	−0.0004 (2)	−0.0017 (2)
S99	0.03358 (11)	0.01552 (8)	0.04409 (14)	−0.00744 (7)	0.01362 (10)	−0.00871 (8)
C98	0.0444 (6)	0.0463 (6)	0.0405 (6)	−0.0221 (5)	0.0041 (5)	−0.0065 (5)
C99	0.0709 (9)	0.0340 (5)	0.0364 (6)	−0.0249 (6)	0.0120 (6)	0.0031 (4)
O99	0.0325 (3)	0.0177 (2)	0.0420 (4)	−0.0088 (2)	0.0113 (3)	−0.0087 (2)
N1'	0.0177 (2)	0.0127 (2)	0.0137 (2)	−0.00486 (17)	0.00368 (17)	−0.00180 (16)
C2'	0.0156 (2)	0.0136 (2)	0.0123 (2)	−0.00444 (18)	0.00174 (18)	−0.00215 (17)
N3'	0.0203 (2)	0.0123 (2)	0.0124 (2)	−0.00580 (17)	0.00448 (18)	−0.00325 (16)
C4'	0.0156 (2)	0.0136 (2)	0.0101 (2)	−0.00448 (18)	0.00092 (18)	−0.00184 (17)
N4'	0.0245 (3)	0.0147 (2)	0.0145 (2)	−0.0079 (2)	0.0066 (2)	−0.00272 (17)
N5'	0.0179 (2)	0.0132 (2)	0.0110 (2)	−0.00406 (17)	0.00235 (17)	−0.00240 (15)
S1'	0.02614 (9)	0.01595 (7)	0.02212 (8)	−0.00640 (6)	0.01245 (7)	−0.00627 (6)
S2'	0.01356 (6)	0.01220 (6)	0.01085 (6)	−0.00175 (4)	0.00147 (4)	−0.00171 (4)
O1'	0.01337 (19)	0.0212 (2)	0.0173 (2)	−0.00446 (16)	0.00224 (16)	−0.00206 (17)
O2'	0.0230 (2)	0.01190 (18)	0.0162 (2)	−0.00003 (16)	0.00084 (17)	−0.00152 (15)
C11'	0.0147 (2)	0.0128 (2)	0.0110 (2)	−0.00346 (18)	0.00122 (18)	−0.00132 (17)
C12'	0.0190 (3)	0.0170 (2)	0.0136 (2)	−0.0074 (2)	−0.0007 (2)	0.00141 (19)
C13'	0.0191 (3)	0.0202 (3)	0.0168 (3)	−0.0084 (2)	−0.0024 (2)	0.0010 (2)
C14'	0.0194 (3)	0.0163 (2)	0.0138 (2)	−0.0032 (2)	−0.0023 (2)	−0.00035 (19)
Cl1'	0.02542 (8)	0.02643 (8)	0.01907 (8)	−0.00473 (6)	−0.00805 (6)	0.00264 (6)
C15'	0.0228 (3)	0.0171 (3)	0.0150 (3)	−0.0068 (2)	−0.0013 (2)	0.0029 (2)
C16'	0.0186 (3)	0.0166 (2)	0.0140 (2)	−0.0069 (2)	0.0004 (2)	0.00121 (19)
C21'	0.0135 (2)	0.0123 (2)	0.0153 (2)	−0.00279 (17)	0.00185 (18)	−0.00025 (18)
C22'	0.0185 (3)	0.0195 (3)	0.0274 (4)	−0.0073 (2)	−0.0044 (3)	0.0020 (2)
C23'	0.0194 (3)	0.0200 (3)	0.0435 (5)	−0.0091 (3)	0.0018 (3)	0.0019 (3)
C24'	0.0254 (4)	0.0155 (3)	0.0361 (4)	−0.0045 (2)	0.0121 (3)	0.0034 (3)
C25'	0.0294 (4)	0.0195 (3)	0.0203 (3)	−0.0019 (3)	0.0047 (3)	0.0053 (2)
C26'	0.0178 (3)	0.0180 (3)	0.0167 (3)	−0.0025 (2)	0.0002 (2)	0.0015 (2)
S98	0.01807 (7)	0.01452 (6)	0.01662 (7)	−0.00496 (5)	0.00397 (5)	−0.00383 (5)
C96	0.0309 (4)	0.0376 (4)	0.0201 (3)	−0.0194 (3)	0.0104 (3)	−0.0118 (3)
C97	0.0306 (4)	0.0142 (3)	0.0340 (4)	−0.0049 (3)	0.0031 (3)	−0.0044 (3)
O98	0.0311 (3)	0.0171 (2)	0.0169 (2)	−0.0090 (2)	0.0028 (2)	−0.00575 (17)

### 1-{N'-[(4-Chlorobenzene)sulfonyl]carbamimidoyl}-3-phenylthiourea dimethyl sulfoxide monosolvate Geometric parameters (Å, º)

**Table:**

N1—C2	1.3338 (9)	C21'—C22'	1.3889 (10)
N1—C21	1.4293 (9)	C22'—C23'	1.3931 (12)
C2—N3	1.3881 (9)	C23'—C24'	1.3866 (15)
C2—S1	1.6746 (7)	C24'—C25'	1.3875 (14)
N3—C4	1.3770 (9)	C25'—C26'	1.3910 (11)
C4—N5	1.3235 (9)	S98—O98	1.5218 (6)
C4—N4	1.3333 (9)	S98—C97	1.7828 (8)
N5—S2	1.6136 (6)	S98—C96	1.7839 (8)
S2—O2	1.4400 (6)	N1—H01	0.848 (15)
S2—O1	1.4463 (6)	N3—H03	0.860 (15)
S2—C11	1.7660 (7)	N4—H041	0.809 (16)
C11—C16	1.3897 (10)	N4—H042	0.861 (15)
C11—C12	1.3967 (9)	C12—H12	0.9500
C12—C13	1.3860 (10)	C13—H13	0.9500
C13—C14	1.3926 (10)	C15—H15	0.9500
C14—C15	1.3856 (11)	C16—H16	0.9500
C14—Cl1	1.7391 (7)	C22—H22	0.9500
C15—C16	1.3979 (11)	C23—H23	0.9500
C21—C22	1.3896 (10)	C24—H24	0.9500
C21—C26	1.3913 (10)	C25—H25	0.9500
C22—C23	1.3948 (11)	C26—H26	0.9500
C23—C24	1.3915 (13)	C98—H98A	0.9800
C24—C25	1.3918 (12)	C98—H98B	0.9800
C25—C26	1.3905 (11)	C98—H98C	0.9800
S99—O99	1.5147 (7)	C99—H99A	0.9800
S99—C98	1.7766 (14)	C99—H99B	0.9800
S99—C99	1.7896 (13)	C99—H99C	0.9800
N1'—C2'	1.3315 (9)	N1'—H01'	0.879 (15)
N1'—C21'	1.4305 (9)	N3'—H03'	0.850 (13)
C2'—N3'	1.3871 (9)	N4'—H04'	0.829 (15)
C2'—S1'	1.6743 (7)	N4'—H04"	0.803 (14)
N3'—C4'	1.3780 (9)	C12'—H12'	0.9500
C4'—N5'	1.3319 (8)	C13'—H13'	0.9500
C4'—N4'	1.3293 (9)	C15'—H15'	0.9500
N5'—S2'	1.6100 (6)	C16'—H16'	0.9500
S2'—O2'	1.4425 (5)	C22'—H22'	0.9500
S2'—O1'	1.4459 (6)	C23'—H23'	0.9500
S2'—C11'	1.7696 (7)	C24'—H24'	0.9500
C11'—C16'	1.3907 (9)	C25'—H25'	0.9500
C11'—C12'	1.3965 (10)	C26'—H26'	0.9500
C12'—C13'	1.3899 (10)	C96—H96A	0.9800
C13'—C14'	1.3937 (10)	C96—H96B	0.9800
C14'—C15'	1.3834 (11)	C96—H96C	0.9800
C14'—Cl1'	1.7399 (7)	C97—H97A	0.9800
C15'—C16'	1.3933 (10)	C97—H97B	0.9800
C21'—C26'	1.3886 (10)	C97—H97C	0.9800
			
C2—N1—C21	124.14 (6)	C21—N1—H01	120.6 (10)
N1—C2—N3	117.33 (6)	C4—N3—H03	114.3 (10)
N1—C2—S1	125.26 (5)	C2—N3—H03	115.9 (10)
N3—C2—S1	117.41 (5)	C4—N4—H041	121.1 (11)
C4—N3—C2	129.78 (6)	C4—N4—H042	119.7 (10)
N4—C4—N5	127.20 (6)	H041—N4—H042	118.3 (14)
N5—C4—N3	118.66 (6)	C13—C12—H12	120.3
N4—C4—N3	114.13 (6)	C11—C12—H12	120.3
C4—N5—S2	122.03 (5)	C12—C13—H13	120.6
O2—S2—O1	117.55 (4)	C14—C13—H13	120.6
O2—S2—N5	105.32 (3)	C14—C15—H15	120.6
O1—S2—N5	112.67 (3)	C16—C15—H15	120.6
O2—S2—C11	108.15 (3)	C11—C16—H16	120.4
O1—S2—C11	107.30 (4)	C15—C16—H16	120.4
N5—S2—C11	105.11 (3)	C21—C22—H22	120.2
C16—C11—C12	121.55 (6)	C23—C22—H22	120.2
C16—C11—S2	120.02 (5)	C24—C23—H23	120.0
C12—C11—S2	118.40 (5)	C22—C23—H23	120.0
C13—C12—C11	119.36 (6)	C23—C24—H24	119.9
C12—C13—C14	118.84 (6)	C25—C24—H24	119.9
C15—C14—C13	122.30 (6)	C26—C25—H25	119.9
C15—C14—Cl1	119.34 (6)	C24—C25—H25	119.9
C13—C14—Cl1	118.35 (6)	C25—C26—H26	120.3
C14—C15—C16	118.80 (7)	C21—C26—H26	120.3
C11—C16—C15	119.15 (7)	S99—C98—H98A	109.5
C22—C21—C26	120.78 (6)	S99—C98—H98B	109.5
C22—C21—N1	119.23 (6)	H98A—C98—H98B	109.5
C26—C21—N1	119.91 (6)	S99—C98—H98C	109.5
C21—C22—C23	119.55 (7)	H98A—C98—H98C	109.5
C24—C23—C22	119.91 (7)	H98B—C98—H98C	109.5
C23—C24—C25	120.13 (7)	S99—C99—H99A	109.5
C26—C25—C24	120.19 (7)	S99—C99—H99B	109.5
C25—C26—C21	119.43 (7)	H99A—C99—H99B	109.5
O99—S99—C98	105.60 (5)	S99—C99—H99C	109.5
O99—S99—C99	105.59 (5)	H99A—C99—H99C	109.5
C98—S99—C99	98.34 (7)	H99B—C99—H99C	109.5
C2'—N1'—C21'	123.45 (6)	C2'—N1'—H01'	114.0 (10)
N1'—C2'—N3'	117.29 (6)	C21'—N1'—H01'	122.4 (10)
N1'—C2'—S1'	124.72 (5)	C4'—N3'—H03'	117.1 (9)
N3'—C2'—S1'	117.97 (5)	C2'—N3'—H03'	112.8 (9)
C4'—N3'—C2'	130.12 (6)	C4'—N4'—H04'	119.6 (10)
N4'—C4'—N5'	127.34 (6)	C4'—N4'—H04"	117.0 (10)
N5'—C4'—N3'	118.47 (6)	H04'—N4'—H04"	123.1 (14)
N4'—C4'—N3'	114.18 (6)	C13'—C12'—H12'	120.2
C4'—N5'—S2'	121.16 (5)	C11'—C12'—H12'	120.2
O2'—S2'—O1'	117.59 (3)	C12'—C13'—H13'	120.7
O2'—S2'—N5'	106.11 (3)	C14'—C13'—H13'	120.7
O1'—S2'—N5'	112.78 (3)	C14'—C15'—H15'	120.5
O2'—S2'—C11'	107.60 (3)	C16'—C15'—H15'	120.5
O1'—S2'—C11'	106.99 (3)	C11'—C16'—H16'	120.3
N5'—S2'—C11'	104.97 (3)	C15'—C16'—H16'	120.3
C16'—C11'—C12'	121.21 (6)	C21'—C22'—H22'	120.4
C16'—C11'—S2'	119.04 (5)	C23'—C22'—H22'	120.4
C12'—C11'—S2'	119.75 (5)	C24'—C23'—H23'	120.0
C13'—C12'—C11'	119.51 (6)	C22'—C23'—H23'	120.0
C12'—C13'—C14'	118.68 (7)	C23'—C24'—H24'	119.8
C15'—C14'—C13'	122.15 (7)	C25'—C24'—H24'	119.8
C15'—C14'—Cl1'	118.06 (5)	C24'—C25'—H25'	120.0
C13'—C14'—Cl1'	119.78 (6)	C26'—C25'—H25'	120.0
C14'—C15'—C16'	119.05 (6)	C21'—C26'—H26'	120.4
C11'—C16'—C15'	119.35 (6)	C25'—C26'—H26'	120.4
C26'—C21'—C22'	121.14 (7)	S98—C96—H96A	109.5
C26'—C21'—N1'	119.00 (6)	S98—C96—H96B	109.5
C22'—C21'—N1'	119.76 (6)	H96A—C96—H96B	109.5
C21'—C22'—C23'	119.13 (8)	S98—C96—H96C	109.5
C24'—C23'—C22'	120.08 (8)	H96A—C96—H96C	109.5
C23'—C24'—C25'	120.33 (7)	H96B—C96—H96C	109.5
C24'—C25'—C26'	120.09 (8)	S98—C97—H97A	109.5
C21'—C26'—C25'	119.20 (7)	S98—C97—H97B	109.5
O98—S98—C97	105.43 (4)	H97A—C97—H97B	109.5
O98—S98—C96	104.57 (4)	S98—C97—H97C	109.5
C97—S98—C96	98.33 (5)	H97A—C97—H97C	109.5
C2—N1—H01	115.3 (10)	H97B—C97—H97C	109.5
			
C21—N1—C2—N3	177.44 (6)	C21'—N1'—C2'—N3'	−176.36 (6)
C21—N1—C2—S1	−3.12 (10)	C21'—N1'—C2'—S1'	5.30 (10)
N1—C2—N3—C4	0.07 (11)	N1'—C2'—N3'—C4'	−3.68 (11)
S1—C2—N3—C4	−179.41 (6)	S1'—C2'—N3'—C4'	174.78 (6)
C2—N3—C4—N5	0.21 (11)	C2'—N3'—C4'—N4'	−175.96 (7)
C2—N3—C4—N4	179.65 (7)	C2'—N3'—C4'—N5'	2.99 (11)
N4—C4—N5—S2	−7.75 (11)	N4'—C4'—N5'—S2'	10.00 (10)
N3—C4—N5—S2	171.61 (5)	N3'—C4'—N5'—S2'	−168.80 (5)
C4—N5—S2—O2	174.75 (6)	C4'—N5'—S2'—O2'	−171.08 (6)
C4—N5—S2—O1	45.38 (7)	C4'—N5'—S2'—O1'	−40.97 (7)
C4—N5—S2—C11	−71.14 (6)	C4'—N5'—S2'—C11'	75.15 (6)
O2—S2—C11—C16	−122.77 (6)	O2'—S2'—C11'—C16'	137.27 (6)
O1—S2—C11—C16	4.96 (7)	O1'—S2'—C11'—C16'	10.04 (6)
N5—S2—C11—C16	125.11 (6)	N5'—S2'—C11'—C16'	−110.01 (6)
O2—S2—C11—C12	55.49 (6)	O2'—S2'—C11'—C12'	−42.27 (6)
O1—S2—C11—C12	−176.78 (5)	O1'—S2'—C11'—C12'	−169.50 (6)
N5—S2—C11—C12	−56.63 (6)	N5'—S2'—C11'—C12'	70.45 (6)
C16—C11—C12—C13	−0.34 (10)	C16'—C11'—C12'—C13'	−1.26 (11)
S2—C11—C12—C13	−178.57 (5)	S2'—C11'—C12'—C13'	178.27 (6)
C11—C12—C13—C14	−0.29 (10)	C11'—C12'—C13'—C14'	−0.73 (11)
C12—C13—C14—C15	0.73 (11)	C12'—C13'—C14'—C15'	1.75 (12)
C12—C13—C14—Cl1	179.44 (5)	C12'—C13'—C14'—Cl1'	−179.62 (6)
C13—C14—C15—C16	−0.52 (12)	C13'—C14'—C15'—C16'	−0.75 (12)
Cl1—C14—C15—C16	−179.22 (6)	Cl1'—C14'—C15'—C16'	−179.41 (6)
C12—C11—C16—C15	0.55 (11)	C12'—C11'—C16'—C15'	2.27 (11)
S2—C11—C16—C15	178.75 (6)	S2'—C11'—C16'—C15'	−177.27 (6)
C14—C15—C16—C11	−0.12 (12)	C14'—C15'—C16'—C11'	−1.25 (11)
C2—N1—C21—C22	110.41 (8)	C2'—N1'—C21'—C22'	−101.83 (9)
C2—N1—C21—C26	−72.77 (9)	C2'—N1'—C21'—C26'	81.93 (9)
C26—C21—C22—C23	0.89 (11)	C26'—C21'—C22'—C23'	−1.48 (12)
N1—C21—C22—C23	177.69 (7)	N1'—C21'—C22'—C23'	−177.63 (7)
C21—C22—C23—C24	−0.54 (12)	C21'—C22'—C23'—C24'	0.41 (13)
C22—C23—C24—C25	−0.14 (12)	C22'—C23'—C24'—C25'	1.15 (14)
C23—C24—C25—C26	0.48 (12)	C23'—C24'—C25'—C26'	−1.68 (13)
C24—C25—C26—C21	−0.14 (11)	C22'—C21'—C26'—C25'	0.97 (11)
C22—C21—C26—C25	−0.55 (11)	N1'—C21'—C26'—C25'	177.14 (7)
N1—C21—C26—C25	−177.33 (7)	C24'—C25'—C26'—C21'	0.62 (12)

### 1-{N'-[(4-Chlorobenzene)sulfonyl]carbamimidoyl}-3-phenylthiourea dimethyl sulfoxide monosolvate Hydrogen-bond geometry (Å, º)

**Table:**

*D*—H···*A*	*D*—H	H···*A*	*D*···*A*	*D*—H···*A*
N1—H01···N5	0.848 (15)	1.929 (15)	2.6375 (8)	140.3 (13)
N3—H03···O99	0.860 (15)	1.902 (15)	2.7300 (9)	161.2 (15)
N4—H041···O1	0.809 (16)	2.341 (16)	2.9130 (10)	128.4 (13)
N4—H041···O2′	0.809 (16)	2.478 (15)	2.9982 (8)	123.2 (13)
N4—H042···O99	0.861 (15)	2.112 (15)	2.8713 (10)	146.7 (13)
N1′—H01′···N5′	0.879 (15)	1.902 (15)	2.6458 (8)	141.3 (14)
N3′—H03′···O98	0.850 (13)	2.060 (13)	2.8379 (8)	151.8 (12)
N4′—H04′···O2^i^	0.829 (15)	2.402 (15)	3.0350 (9)	133.8 (13)
N4′—H04′···O1′	0.829 (15)	2.220 (15)	2.8324 (9)	130.9 (13)
N4′—H04"···O98	0.803 (14)	2.035 (14)	2.7903 (9)	156.7 (14)
C13—H13···S1^ii^	0.95	3.01	3.6616 (7)	127
C15—H15···S1′^iii^	0.95	2.80	3.6671 (8)	153
C98—H98*C*···O1′	0.98	2.66	3.4937 (15)	144
C99—H99*C*···S1	0.98	3.01	3.8460 (13)	144
C15′—H15′···S1^iv^	0.95	3.02	3.9409 (7)	165
C16′—H16′···O2^i^	0.95	2.45	3.3273 (9)	153
C26′—H26′···O2^v^	0.95	2.55	3.2038 (9)	127
C96—H96*B*···O1^vi^	0.98	2.60	3.2186 (11)	121
C97—H97*C*···O2′^i^	0.98	2.56	3.4130 (11)	146

Symmetry codes: (i) *x*, *y*−1, *z*; (ii) −*x*+1, −*y*+2, −*z*+2; (iii) −*x*+2, −*y*+1, −*z*+1; (iv) −*x*+1, −*y*+1, −*z*+2; (v) −*x*+1, −*y*+2, −*z*+1; (vi) −*x*+1, −*y*+1, −*z*+1.
